# Association between Tomographic Characteristics of Pterygium and Preoperative Anterior and Posterior Topography Measured by Anterior Segment Optical Coherence Tomography

**DOI:** 10.3390/life14101245

**Published:** 2024-09-29

**Authors:** Marina Aguilar-González, Enrique España-Gregori, Isabel Pascual-Camps, M. Dolores Pinazo-Durán, Cristina Peris-Martínez

**Affiliations:** 1 Unit of Cornea and Anterior Eye Diseases, Fundación de Oftalmología Médica (FOM), C/Pío Baroja 12, 46015 Valencia, Spain; cristinaperismartinez0@gmail.com; 2 Hospital de Manises, Av. de la Generalitat Valenciana 50, 46940 Manises, Spain; 3 Hospital Universitario y Politécnico La Fe, Av. Fernando Abril Martorell 106, 46026 Valencia, Spain; enrique.espana@uv.es (E.E.-G.); i.pascualcamps@gmail.com (I.P.-C.); 4 Department of Surgery, Ophthalmology, Universitat de Valencia, Avenida Blasco Ibáñez 15, 46010 Valencia, Spain; dolores.pinazo@uv.es; 5 Aviñó Peris Eye Clinic, Avenida del Oeste 34, 46001 Valencia, Spain

**Keywords:** pterygium, ocular surface imaging, anterior segment ocular coherence tomography, AS-OCT, astigmatism, corneal topography

## Abstract

**Background:** The utilities of anterior segment optical coherence tomography (AS-OCT) for characterization, differential diagnosis, postoperative monitoring, and evaluation/comparison of surgical techniques in pterygium are described. Through AS-OCT, it is also possible to study the corneal astigmatic effect of pterygium. Our purpose is to study the associations between the anatomical characteristics of pterygium and the corneal topography through AS-OCT. **Methods:** Fifty eyes with primary pterygium in a tertiary hospital were evaluated before surgery by measuring 10 anatomical variables of pterygium and 13 topographic variables using AS-OCT (Casia 2; Tomey Corp., Nagoya, Japan). Statistical analysis was used to study the association between them. **Results:** Pterygium classified as flat pattern exhibited lower preoperative values of flat keratometry (K1), real flat keratometry (K1r), average keratometry (AvgK), and real average keratometry (AvgKr) compared to nodular ones. The flat pattern showed greater cylinder (CYL) and real cylinder (CLYr) values. The horizontal corneal invasion proportionally increased CYL and CYLr. Overall, larger anatomical pterygium measurements (limbus thickness (LimbusT), central pterygium thickness (CentreT), head pterygium thickness (HeadT), epithelial thickness at 1 mm (EpitT1mm), stromal thickness at 1 mm (stromT1mm), total thickness at 1 mm (TotalT1mm), total thickness at 2 mm (TotalT2mm), and total thickness at 3 mm (TotalT3mm)) resulted in lower anterior K1, K1r, AvgK, and AvgKr, and posterior K1 and AvgK values. CentreT was greater in astigmatisms against the rule than in oblique ones. **Conclusions:** This study demonstrates associations between preoperative topography and the NF (nodular or flat) classification of pterygium and its anatomical measurements assessed by AS-OCT.

## 1. Introduction

Anterior segment optical coherence tomography (AS-OCT) for ocular surface examination is a relatively new imaging method that allows for real-time, artifact-free, high-resolution quantitative imaging of biological tissue structures in the anterior segment of the eye in a reliable, reproducible, fast, and non-invasive manner. It enables the assessment of anatomical tissue differences with multiple diagnostic, prognostic, and therapeutic applications [1,2,3]. Pterygium, as a lesion on the ocular surface, can be studied more accurately and easily reproducible using AS-OCT than with slit-lamp examination (SLE) [4], and the utility of AS-OCT for its characterization, differential diagnosis, postoperative monitoring, and evaluation/comparison of surgical techniques has been described [5,6,7,8]. Furthermore, AS-OCT allows for the study of these anatomical characteristics of pterygium while simultaneously performing corneal topography. This makes it easier to analyze the association of anatomical aspects of the lesion with corneal astigmatism with a single test.

Our hypothesis is that some anatomical features of pterygium, which are measurable with AS-OCT but not discernible with SLE, are associated with topographic values. The purpose of this study is to evaluate the influence of pterygium characteristics in the preoperative anterior and posterior corneal topography, determining whether there is an association between the NF (nodular or flat) classification of the pterygium or its anatomical measurements (limbus thickness, central thickness, head thickness, horizontal corneal invasion, epithelial thickness at 1 mm, stromal thickness at 1 mm, total thickness at 1 mm, total thickness at 2 mm, and total thickness at 3 mm) and preoperative topographic values by AS-OCT. Additionally, we also assess whether there is an association between AIF (atrophic, intermediate, or fleshy) classification by SLE and preoperative topography in order to compare the relative value of the AS-OCT (NF) and SLE (AIF) classifications. The main conclusion of this study is that there are associations between preoperative topography, including posterior topography, and the pterygium tomographic classification (nodular or flat) and its anatomical measurements assessed only by AS-OCT, which are not assessable with SLE.

## 2. Materials and Methods

This project was approved by the Institution’s Research Ethics Committee (Drug Research Ethics Committee of the CEI—Hospital Universitario y Politécnico La Fe, date of approval 10 August 2020 and the CEI—Fundación de Oftalmología Médica de la Comunidad Valencina (FOM), date of approval 30 September 2021) with the trial numbers 2020-294-1 and PI 107, respectively. Every patient gave their informed consent in accordance with the Declaration of Helsinki.

### 2.1. Inclusion and Exclusion Criteria

Fifty eyes of fifty patients with a surgical indication for primary pterygium at a tertiary ophthalmology center were included. Patients with other relevant corneal or conjunctival pathologies unrelated to pterygium that could influence corneal topography, ocular treatments (excluding artificial tears) lasting longer than 1 month, prior ocular surgery, recurrent pterygium, or patients under 18/over 70 years of age were excluded.

### 2.2. Clinical Assessment Protocol

Patients were classified according to the AIF pattern as atrophic (vascularization below the body of the pterygium), intermediate (intermediate characteristics between the other two), or fleshy (vascularization in the body of the pterygium) with SLE. They were also classified according to the NF pattern as nodular (when the subepithelial mass causes a convex change in the curvature of the ocular surface at the limbus) [9] or flat/continuous (the shape of the ocular surface contour is not altered, and there is a hypodense space with a “void” appearance in the sclerocorneal angle) with AS-OCT [10]. In all patients, 10 pterygium anatomical variables and 13 corneal topography variables were measured with AS-OCT (Casia 2; Tomey Corp., Nagoya, Japan) with a protocol designed specifically for this study called “PTERYGIUM PROTOCOL”, which includes standard topography (keratometric, posterior, and real maps) and manual measurement of pterygium’s anatomical characteristics (2D analysis).

**Figure 1:**
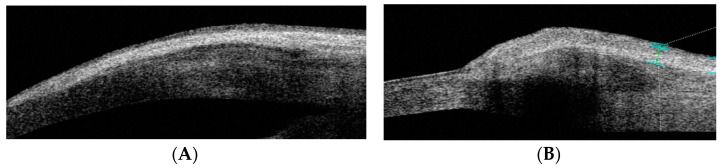
AS-OCT image of the tomographic classification of pterygium as flat or nodular. (**A**) OCT-SA image of flat pterygium: the shape of the ocular surface contour is not altered. (**B**) OCT-SA image of flat pterygium: the subepithelial mass of the pterygium is seen to cause a convex change in the curvature of the ocular surface of the limbus.

The following 10 pterygium anatomical parameters were measured in millimeters using AS-OCT: limbus thickness (LimbusT), central pterygium thickness (CentreT), head pterygium thickness (HeadT), horizontal corneal invasion (Horizontal Corneal Inv), epithelial thickness at 1 mm (EpitT1mm), stromal thickness at 1 mm (stromT1mm), total thickness at 1 mm (TotalT1mm), total thickness at 2 mm (TotalT2mm), total thickness at 3 mm (TotalT3mm), and pterygium type (nodular or flat) (Figure 1).

**Figure 2:**
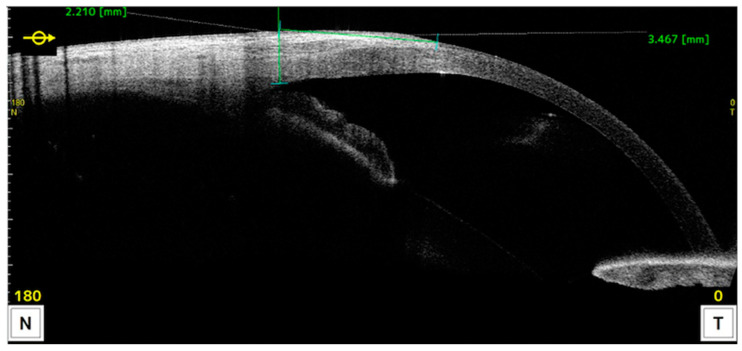
AS-OCT image illustrating the measurement of corneal invasion. The reference point is taken by drawing a line perpendicular to the ocular surface starting at the scleral spur.

**Figure 3:**
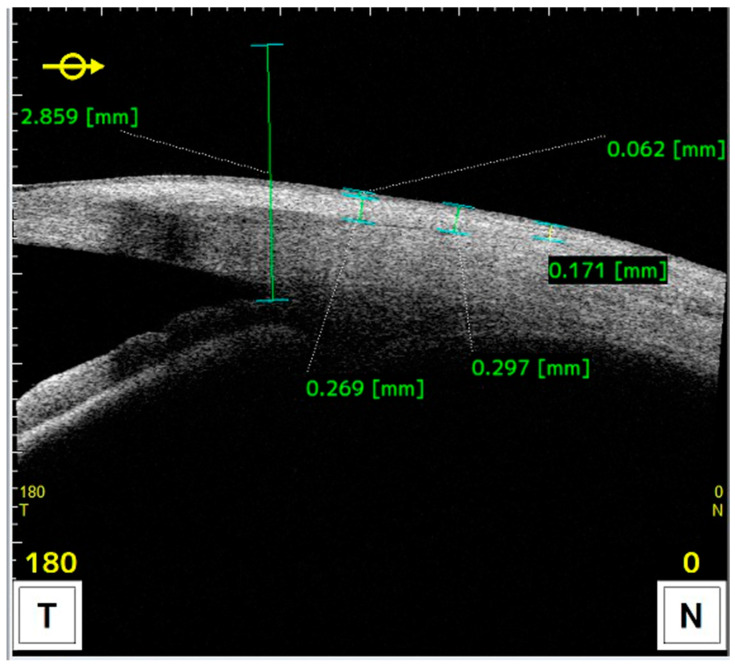
AS-OCT image illustrating measurements taken towards the conjunctiva at 1, 2, and 3 millimeters from the reference point.

Measurements of anatomical characteristics using AS-OCT were made in the horizontal meridian at the center of the pterygium as follows (as described in previous studies) (Figure 2 and Figure 3) [6]:

1. Drawing a line perpendicular to the ocular surface at the scleral spur level.
2. The intersection of this line with the ocular surface is the reference point from which measurements were taken towards the cornea or towards the bulbar conjunctiva or graft.

The following 13 corneal topography variables were measured using AS-OCT with the axial power topography map:

- Keratometry: corneal astigmatism power (CYL), flat keratometry (K1), steep keratometry (K2), mean keratometry (AvgK);
- Posterior Keratometry: posterior corneal surface astigmatism power (CYLp), posterior flat keratometry (K1p), posterior steep keratometry (K2p), mean posterior keratometry (AvgKp);
- Real Keratometry: real astigmatism (CYLr), real flat keratometry (K1r), real steep keratometry (K2r), mean real keratometry (AvgKr).
- Type of astigmatism:
  o With-the-rule astigmatism: when the meridian with the highest dioptric power or the most curved is the vertical (K2 of 90 ± 20), and the axis of the cylinder is at 0 ± 20.
  o Against-the-rule astigmatism: when the meridian with the highest dioptric power or the most curved is the horizontal (K2 180 ± 20), and the axis of the cylinder is at 90 ± 20.
  o Oblique astigmatism: when the maximum curvature of the cornea is not found on either of the horizontal or vertical meridians but at an oblique angle (none of the K2 values mentioned above).

All variables were measured by the same researcher and within the same time frame (between 9 a.m. and 2 p.m.).

### 2.3. Statistical Analysis

Statistical analysis was conducted using the SPSS program (SPSS Statistics^®^, IBM^®^, Armonk, NY, USA, version 21.0.0.0) for descriptive variables and R-project (The R foundation©, version 3.0.2) for other analyses.

The approach to sample size calculation is applicable to the detection of correlation between continuous variables (degree of correlation between anatomical variables and astigmatism). In Table 1, we can see the total sample size (number of eyes) needed to detect a certain effect size in the degree of correlation between two continuous parameters, calculated for 3 levels of statistical power (95% confidence level with Pearson’s linear correlation coefficient null test).

In other words, to detect a weak-moderate correlation (r = 0.4) as statistically significant with a power of 80%, a minimum of 46 patients would be needed. In other words, with the 50 patients finally enrolled in this study, a power of 80% was achieved to detect a weak-moderate correlation (r = 0.4) at the 95% confidence level.

The astigmatism variables (CYL, K1, K2, AvgK, CYLp, K1p, K2p, AvgKp, CYLr, K1r, K2r, AvgKr, and K2 axis) and preoperative anatomical features of pterygium (LimbusT, CentreT, HeadT, Horizontal Corneal Inv, EpitT 1 mm, stromT 1mm, TotalT 1 mm, TotalT 2mm, and TotalT 3mm) measured by AS-OCT were the primary variables of this research. The sample size was 50 eyes.

The Kolmogorov–Smirnov test indicated occasional deviations from normality in some parameters. Given these premises, the general approach to the analysis was parametric. However, non-parametric tests were also conducted in situations of deviation to ensure the consistency and reliability of the results. The non-parametric tests equivalent to those performed were the Mann–Whitney test, the Wilcoxon test, the Kruskal–Wallis test, and Spearman’s correlation. The parametric statistical tests used included the independent samples *t*-test to compare the equality of mean values in two independent groups of patients, the ANOVA (F-Snedecor statistic) to compare the equality of means in more than two independent samples, Pearson’s R correlation to evaluate the linear association between two continuous variables, the chi-squared test to measure the degree of association between two categorical variables (with consideration of Fisher’s exact test when expected cell frequencies were too low), the binomial test to determine if the proportion of stable astigmatisms was significantly different from an expected or theoretical proportion, and simple linear regression to establish a functional relationship between two variables.

The significance level used in the analyses was set at 5% (α = 0.05)

## 3. Results

### 3.1. Descriptive Analysis

The research sample consisted of 50 eyes from 50 patients. Half of the patients were men and half were women, with a mean age of 43 ± 9.5 years and a range of 27 to 61 years; 60% of the eyes had an intermediate pattern, 32% had a fleshy pattern, and 8% had an atrophic pattern (this last one, given its small number, was excluded from statistical tests). A higher frequency of nodular pattern (62%) was observed compared to the flat pattern (38%); 98% of the eyes had nasal pterygium, while 2% had pterygium in both nasal and temporal locations; 52% of the eyes had unilateral pterygium, and 48% had bilateral pterygium.

### 3.2. Inferential Analysis

Regarding the association between the type of pterygium measured with SLE (AIF pattern) and preoperative topographic values, no significant differences were found for any astigmatism variable based on the AIF pattern (*p* > 0.05) (Table 2).

Regarding the association between the type of pterygium measured with SLE (AIF pattern) and the type of astigmatism (with-the-rule, against-the-rule, or oblique), no statistically significant differences were observed in the type of astigmatism with respect to the AIF pattern in slit lamp (*p* = 0.815) (Table 3).

Regarding the association between the type of pterygium measured with AS-OCT (NF pattern) and preoperative topographic values, statistically significant differences were detected between the NF pattern and four of the astigmatism variables. Regarding other parameters, there were two in which some trends were observed (Table 4).

- K1

**Figure 4:**
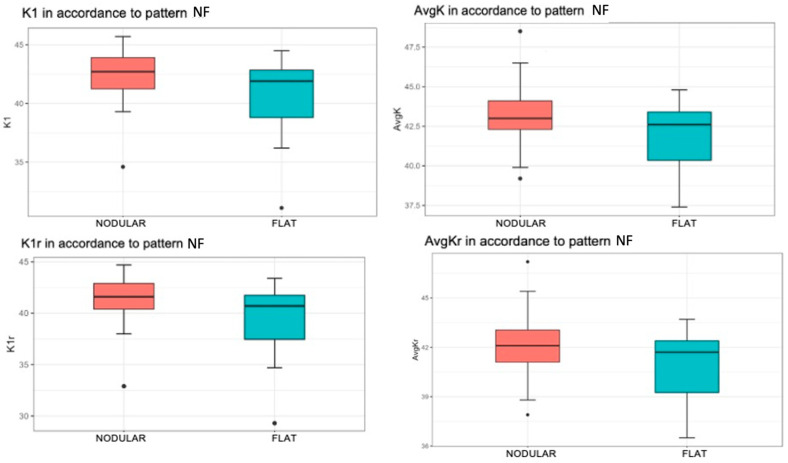
Associations of keratometry and true keratometry with the nodular/flat pterygium pattern measured with OCT-SA Red color represents nodular pattern and blue color represents flat pattern. K1 by nodular/flat pattern (**top left**). The box represents 50% of the cases, with the median as the horizontal line dividing it. The upper and lower edges of the box correspond to the 1st and 3rd quartiles, below which are 25% and 75% of the sample, respectively. The “whiskers” extend to values within an acceptable range, above which are outliers (circles) and extremes (asterisks). AvgK by nodular/flat pattern (**top right**). K1r by nodular/flat pattern (**bottom left**). AvgKr by nodular/flat pattern (**bottom right**).

A significantly higher mean value of K1 was found when the patient had a nodular pattern (*p* = 0.025). Specifically, the mean value of the K1 variable was 42.36 for patients with a nodular pattern, compared to the flat pattern with a mean value of 40.31 (Figure 4, top left).

- AvgK

There was a statistically significant increase in the mean value of AvgK when the patient exhibited a nodular pattern (*p* = 0.028). The mean value of AvgK was 43.30 for patients with a nodular pattern and 41.96 for patients with flat pattern (Figure 4, top right).

- K1r

A statistically significant difference in the mean value of K1r was observed with respect to the NF pattern (*p* = 0.022). More specifically, this mean value was 41.33 in patients with a nodular pattern and 39.09 in patients with flat pattern (Figure 4, bottom left).

- AvgKr

When the patient had a nodular pattern, there was a statistically significant increase in the mean value of the AvgKr variable (*p* = 0.024). That is, the mean value was 42.23 when the patient had a nodular pattern, while the mean value was 40.85 if the patient had a flat pattern (Figure 4, bottom right).

In terms of trends, there was a moderate difference in the CYL variable with respect to different NF pattern types (*p* = 0.072), and a strong trend was detected between the CYLr variable and this pattern (*p* = 0.060). In both parameters, the mean was more negative in flat patterns.

When studying the association between the type of pterygium measured with AS-OCT (NF pattern) and the type of astigmatism (with-the-rule, against-the-rule, or oblique), there was no statistical evidence to determine that the NF pattern type is different with respect to any type of axis astigmatism (*p* = 0.885) (Table 5).

**Figure 5:**
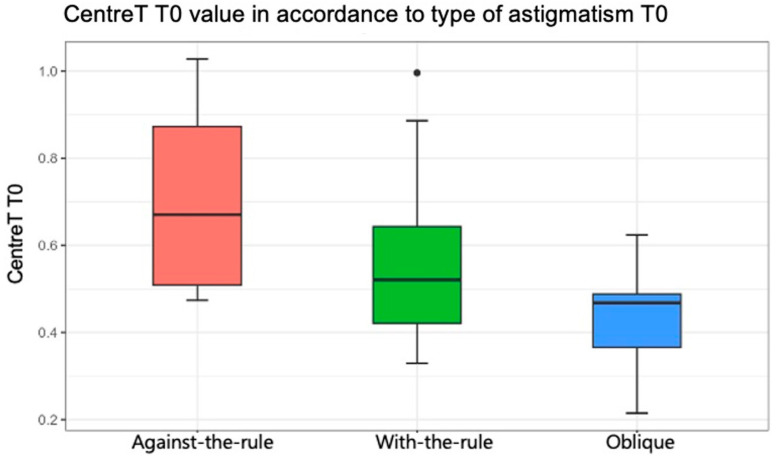
Statistically significant association between the variable “Center Thickness of Pterygium” and the axis of astigmatism (*p* = 0.047). Red color represents against-the-rule astigmatism, green color represents with-the-rule astigmatism and blue color represent olique astigmatism.

Regarding the association between the anatomical characteristics of pterygium measured with AS-OCT (LimbusT, CentreT, HeadT, Horizontal Corneal Inv, EpitT1 mm, stromT1mm, TotalT1mm, TotalT2mm, and TotalT3mm) and the type of astigmatism (with-the-rule, against-the-rule, or oblique), Table 6 shows the comparison of thickness parameters by preoperative astigmatism type (result of ANOVA test). In general, there was no statistical evidence that the pterygium measurements were associated with a type of astigmatism pattern (*p* > 0.05), except for the CentreT measurement (*p* = 0.047). The mean values for CentreT were 0.55, 0.71, and 0.43 when the horizontal meridian of the cornea was more curved (with-the-rule astigmatism), when the vertical meridian was more curved (against-the-rule), and oblique, respectively (Figure 5). In Table 7, multiple comparisons of CentreT by type of astigmatism are shown (results of *t*-tests with Bonferroni correction). It was found that there were only statistically significant differences between the value of CentreT when the type of astigmatism was against-the-rule and oblique (*p* = 0.042). Indeed, the mean value of the CentreT variable was 0.71 when astigmatism was against-the-rule, and this mean value decreased to 0.43 when the astigmatism was oblique.

Regarding the association between the anatomical features of pterygium measured with AS-OCT (LimbusT, CentreT, HeadT, Horizontal Corneal Inv, EpitT1mm, StromT1mm, TotalT1mm, TotalT2mm, and TotalT3mm) and preoperative topographic values, Table 8 shows the relationship between astigmatism parameters and measurements of preoperative pterygium thickness (Pearson correlation coefficient).

- CYL

**Figure 6:**
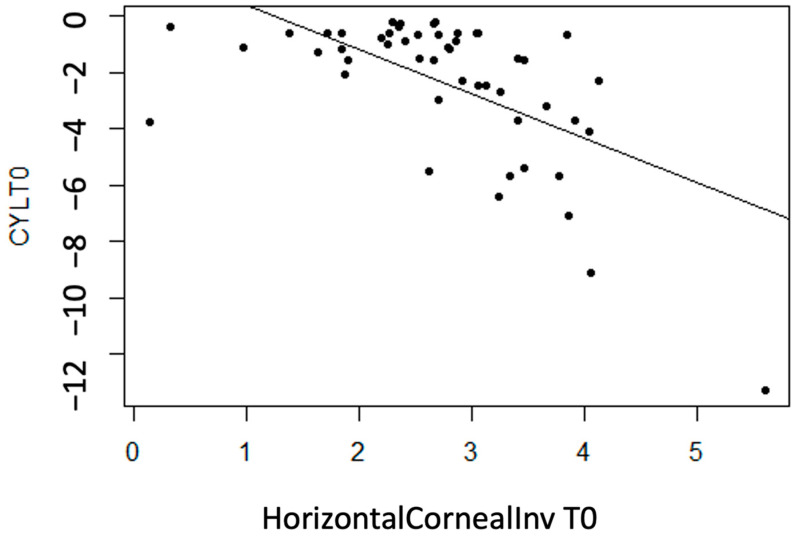
Moderate inverse association between the variables “CYL at T0” and “Horizontal Corneal Invasion at T0”.

No linear statistically significant association is observed for regarding thickness measurement variables (*p* > 0.05), except for InvCorneaHoriz (*p* < 0.001). It is concluded that there is a moderate inverse association between the variables (r = −0.60), meaning that with more horizontal corneal invasion of the pterygium, the CYL value tends to become more negative (astigmatism increases) (Figure 6).

- K1

**Figure 7:**
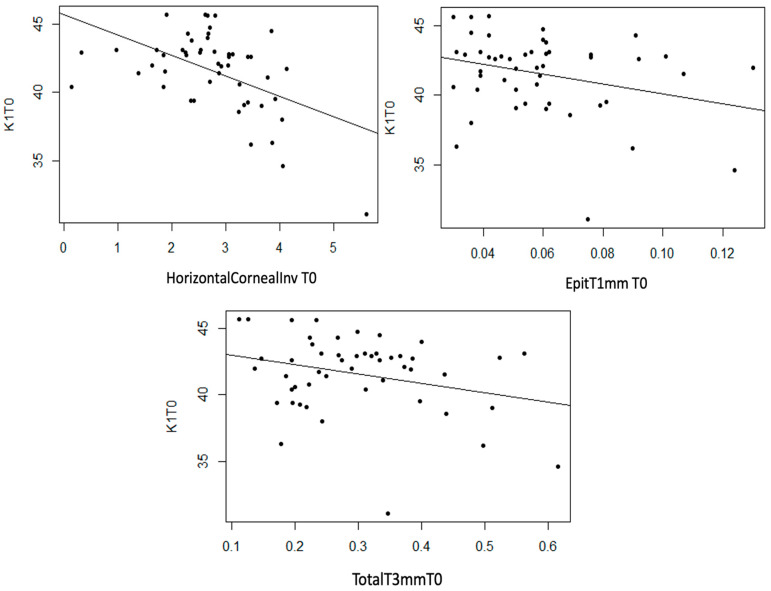
Moderate inverse association between the variables “Horizontal Corneal Invasion at T0”, “Total Thickness at 3 mm at T0”, and “Epithelial Thickness at 1 mm at T0” with the variable “K1 at T0”.

Statistically significant linear associations were detected between K1 astigmatism and InvCorneaHoriz (*p* < 0.001), EpitT1mm (*p* = 0.040) and TotalT3mm (*p* = 0.049), as well as a strong trend with TotalT2mm (*p* = 0.056). A moderate inverse linear relationship was observed between these three anatomical variables and K1, meaning that as the value of these variables increases, the value of K1 decreases (Figure 7).

- K2

No statistically significant linear relationship was observed for the K2 astigmatism variable and the thickness measurement variables (*p* > 0.05). Nevertheless, StromT1mm and TotalT1mm present a moderate trend with K2 (*p* = 0.073 and *p* = 0.062, respectively).

- AvgK

**Figure 8:**
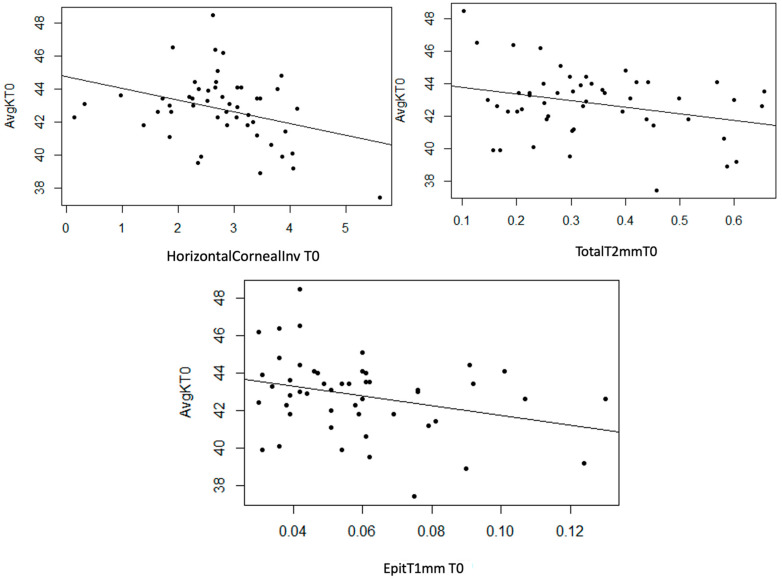
Moderate inverse association between the variables “Horizontal Corneal Invasion at T0”, “Epithelial Thickness at 1 mm at T0”, and “Total Thickness at 2 mm at T0” with the variable “AvgK at T0”.

Statistically significant linear associations were observed between AvgK and InvCorneaHoriz (*p* = 0.017), and EpitT1mm (*p* = 0.035) and TotalT2mm (*p* = 0.041). Furthermore, a strong trend was noted with TotalT1mm (*p* = 0.054) and a moderate trend with StromT1mm (*p* = 0.082) and TotalT3mm (*p* = 0.071) variables. In the three scatter plots, a moderate inverse linear relationship can be observed, as an increase in the values of the mentioned thickness variables leads to a decrease in AvgK (Figure 8).

- CYLp

No statistically significant linear relationship is detected for the CYLp astigmatism variable regarding thickness measurement variables (*p* > 0.05).

- K1p

**Figure 9:**
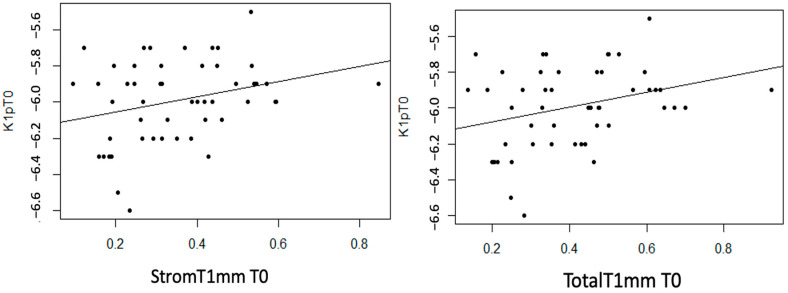
Moderate direct association between the variables “Stromal Thickness at 1 mm at T0” and “Total Thickness at 1 mm at T0” with the variable “K1p at T0”.

StromT1mm and TotalT1mm show a statistically non-null linear correlation with K1p (*p* = 0.044 and 0.032, respectively). Additionally, a moderate trend was observed with the EpitT1mm variable (*p* = 0.077). In both cases, a moderate direct linear correlation was observed, meaning that as the thickness variable value increases, K1p becomes more positive (decreases) (Figure 9).

- K2p

No case was considered where a statistically significant linear relationship was detected (*p* > 0.05). However, a moderate trend was observed with EpitT1mm (*p* = 0.052).

- AvgKp

**Figure 10:**
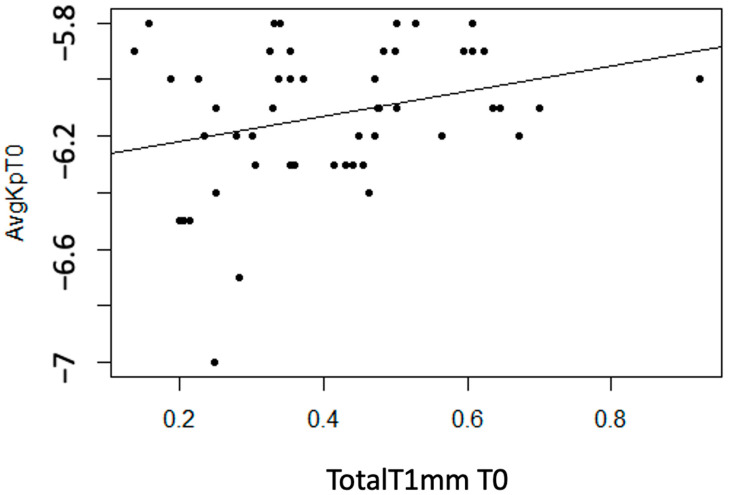
Moderate direct association between the variable “Total Thickness at 1 mm at T0” and “AvgKp at T0”.

Statistically significant linear associations were observed between AvgKp and TotalT1mm (*p* = 0.042). Additionally, a strong trend was found with StromT1mm (*p* = 0.055). A moderate direct linear association was observed, meaning that as the thickness variable value increases, the AvgKp variable becomes more positive (decreases) (Figure 10).

- CYLr

**Figure 11:**
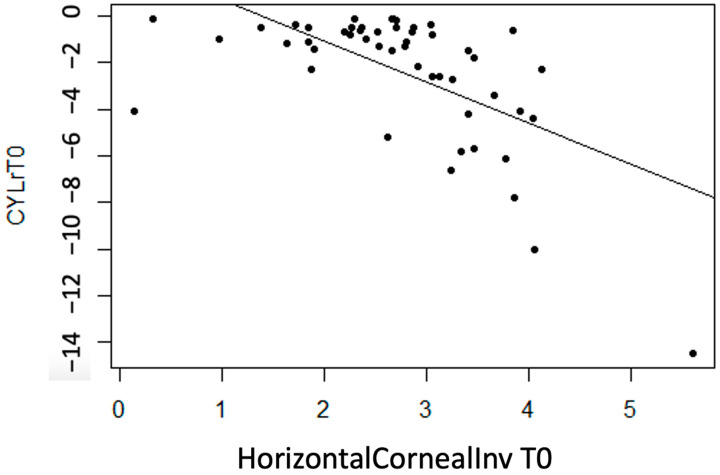
Moderate inverse association between the variables “Horizontal Corneal Invasion at T0” and “CYLr at T0”.

No statistically significant linear correlation is considered for the CYLr astigmatism variable regarding thickness measurement variables (*p* > 0.05), except for InvCorneaHoriz (*p* < 0.001). A moderate linear correlation is detected between InvCorneaHoriz and CYLr, concluding that as the horizontal corneal invasion variable value increases, CYLr value becomes more negative (Figure 11).

- K1r

**Figure 12:**
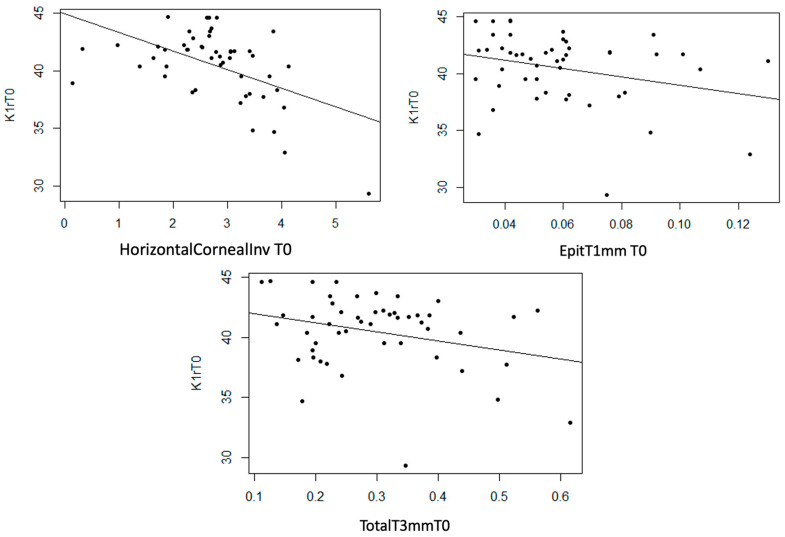
Moderate inverse association between the variables “Horizontal Corneal Invasion at T0”, “Epithelial Thickness at 1 mm at T0”, and “Total Thickness at 3 mm at T0” with the variable “K1r at T0”.

InvCorneaHoriz, EpitT1mm, and TotalT3mm variables show a statistically non-null linear relationship with K1r (*p* < 0.001, *p* = 0.046, and *p* = 0.044, respectively). Additionally, a strong trend was observed with StromT1mm (*p* = 0.055). In the three scatter plots, a moderate inverse linear relationship was observed, meaning that as the thickness variable values increase, K1r values decrease (Figure 12).

- K2r

No statistically non-null linear relationship was considered for the thickness measurement variables (*p* > 0.05), although a moderate trend was observed in StromT1mm (*p* = 0.085) and TotalT1mm (*p* = 0.077) variables.

- AvgKr

**Figure 13:**
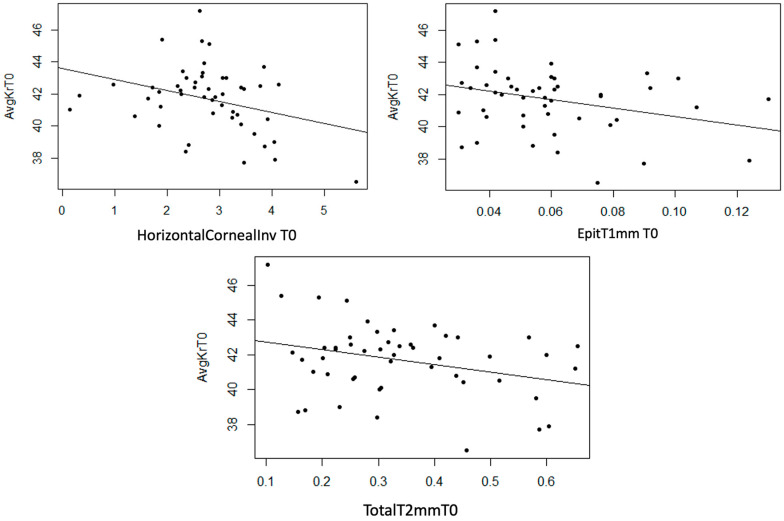
Moderate inverse association between the variables “Horizontal Corneal Invasion at T0”, “Epithelial Thickness at 1 mm at T0”, and “Total Thickness at 2 mm at T0” with the variable “AvgKr at T0”.

Statistically significant linear correlations were observed between AvgKr and InvCorneaHoriz (*p* = 0.022), EpitT1mm (*p* = 0.033), and TotalT2mm (*p* = 0.032) variables. Furthermore, a strong trend was observed in TotalT1mm (*p* = 0.053) and TotalT3mm (*p* = 0.056) variables, and a moderate trend was observed in the StromT1mm (*p* = 0.081). In the three scatter plots, a moderate inverse linear correlation can be observed, meaning that as the thickness variables increase, AvgKr decreases (Figure 13).

## 4. Discussion

With the introduction of AS-OCT as a tool for studying the ocular surface, multiple applications of its use in pterygium have been published. These applications include the possibility of a differential diagnosis with other ocular surface pathologies [10,11,12,13], the study of the influence of this lesion and surgery on corneal topography [14,15,16,17,18,19,20,21,22], the anatomical knowledge of the lesion that allows differentiation between different pterygium patterns with different behaviors and prognoses [4,5,10,23], knowledge of various factors that could lead to recurrence [10,24,25,26,27], anatomical monitoring of graft status after excision surgery with conjunctival autograft or other techniques, and comparison between different surgical techniques [5,6,7,8].

Focusing on the study of the relationship between pterygium and corneal astigmatism, it is known that pterygium, as a corneal surface lesion, induces changes in corneal topography [14,16,18]. Due to its location, the topographic alterations on the anterior corneal surface have been well described. However, due to its immersion in the corneal stroma, it is expected to also alter the posterior corneal surface, and these alterations may be influenced by the size and other anatomical characteristics of the lesion. In this study, we evaluate the influence of pterygium and its anatomical measurements on corneal topography, including both the anterior and posterior surfaces, as the posterior corneal surface in pterygium has been under-studied. Furthermore, in most of the published papers, AS-OCT is either not used for data acquisition or is only used for taking some measurements, with pterygium characteristics and astigmatism being measured using different techniques (refraction, retinoscopy, autorefractometer, pathological anatomy, LH, anterior pole photographs, etc.), and that is why one of the objectives of our study is to combine the collection of topographical and anatomical data of pterygium in a single session with a specific AS-OCT protocol. Next, we compare the results of these previous publications with the findings of our study.

Pterygium have been classified as nodular or flat with AS-OCT previously, and although not statistically significant, it has been observed that anterior corneal astigmatism is greater in those with a flat/continuous pattern [15]. It has also been demonstrated that the reduction in pterygium head thickness and the flat corneoscleral transition zone are associated with an increased corneal stromal scarring (measured by pathological anatomy) and preoperative astigmatism (measured with an autorefractometer) [14]. In our study, in line with previous findings, the flat pterygium pattern exhibited cylinder and real cylinder values with a moderate and strong tendency, respectively, to be higher than the nodular pattern. However, pterygium head thickness was not statistically significantly associated with an increase in preoperative astigmatism. Additionally, our study revealed that the values of K1, K1r, AvgK, and AvgKr significantly lower in a flat pattern, which had not been previously studied. Our work also concludes that the NF pattern does not influence the type of astigmatism. It is worth noting that, in contrast to the topographic differences observed between patterns using AS-OCT classification in the NF pattern, we did not find statistically significant differences between patterns for any preoperative astigmatism variables when classified by LH in the AIF pattern.

The association between anatomical pterygium characteristics measured with AS-OCT (epithelial thickness near the apex (μm), apical or head thickness (μm), limbal thickness (μm), horizontal pterygium length (mm), central corneal thickness (μm), and maximum pterygium thickness (mm)) and preoperative refractive and keratometric astigmatism measured with refraction, retinoscopy, or autorefractometer has been published. A statistically significant association of horizontal pterygium length with keratometric astigmatism and a significant positive correlation of limbal thickness with refractive astigmatism were observed, but not for the rest of the parameters [16]. In our study, in line with the previous findings, horizontal corneal invasion in mm, measured with AS-OCT, was statistically significantly associated with more negative values (increase) in CYL and CYLr and a decrease in K1, K1r, AvgK, and AvgKr. However, unlike the previous studies, limbal thickness was not statistically significantly associated with any preoperative topographic variable, while other anatomical characteristics not described in any previous study were statistically significantly associated. In our study, epithelial thickness at 1 mm and total thickness at 3 mm showed a moderate inverse linear relationship with K1 and K1r, epithelial thickness at 1 mm and total thickness at 2 mm showed a moderate inverse linear relationship with AvgK and AvgKr, and other thickness variables showed association with posterior topography (analyzed later). In summary, our study shows a statistically significant relationship between most anatomical measurements of pterygium (horizontal corneal invasion, epithelial thickness at 1 mm, stromal thickness at 1 mm, total thickness at 1 mm, total thickness at 2 mm, and total thickness at 3 mm), except for three (limbal thickness, center, and head of pterygium), and preoperative topography. It is evident that with larger measurements of pterygium, flat and mean keratometry decrease in anterior topography, which is consistent with the decrease in flat and mean keratometry observed in our study in flat pattern pterygia compared to nodular ones. This is in line with the fact that pterygium lesions exert traction forces in the flat meridian, and these traction forces are greater in flat pattern pterygia. Therefore, it would be interesting to investigate in future studies whether the flat pattern is associated with greater conjunctival thickness. However, none of the anatomical measurements using AS-OCT of pterygium are associated with the type of astigmatism, except for the thickness in the center of the pterygium when the type of astigmatism is against-the-rule and oblique, with this thickness being greater in astigmatism against-the-rule.

Regarding the effect of pterygium on the posterior surface topography, previous studies have examined the effect of surgery on the posterior surface. Some have not observed significant changes [18,22], while others have observed it [19]. Changes in posterior corneal surface have been demonstrated after pterygium excision; thus, we can conclude that pterygium induces a posterior corneal astigmatism effect that is reversed after surgery [19]. This astigmatic effect increases with age and higher preoperative posterior astigmatism, but it has not shown correlation with depth and size of pterygium [19]. In our study, however, several dimensions of pterygium measured with AS-OCT were indeed associated with preoperative posterior topographic variables. Thus, stromal thickness at 1 mm and total thickness at 1 mm were statistically significantly associated with preoperative K1p, and total thickness at 1 mm was associated with preoperative AvgKp, such that, with larger thickness values, flat and mean keratometry of the posterior topography become more positive (decrease), just as in anterior topography.

This work demonstrates that classifying pterygium using AS-OCT in the NF pattern allows for predicting its behavior in topography, unlike the classification using LH. Likewise, the size of pterygium can be more precisely measured with AS-OCT than with LH, and AS-OCT also allows for depth thickness anatomical measurements that cannot be assessed by LH and have shown association with preoperative topography. In general, our findings align with previous studies, and we have described new correlations between anatomical measurements of pterygium and their association with corneal topography, both anterior and posterior, including the following: (1) Flat pattern pterygia are associated with lower values of anterior flat and mid keratometry. (2) Larger anatomical measurements of pterygium, including epithelial thickness at 1 mm and total thickness at 3 mm, are associated with lower values of anterior flat keratometry; larger measurements of epithelial thickness at 1 mm and total thickness at 2 mm are associated with lower values of anterior-mid keratometry. (3) Larger anatomical measurements of pterygium, including stromal thickness at 1 mm and total thickness at 1 mm, are statistically significantly associated with lower (more positive) values of posterior flat and mean keratometry, such as the anterior ones. Moreover, this work is, to the best of our knowledge, the first to study all these associations through a single procedure, AS-OCT.

## 5. Conclusions

Our results showed a statistically significant correlation between some anatomic pterygium variables and some topography variables.

1. The flat pattern of the AS-OCT classification showed lower values of anterior flat and mean keratometry than the nodular pattern, while the SLE did not show differences between patterns.
2. When de horizontal corneal invasion increases, the anterior cylinder increases in the same way.
3. When most of the depth pterygium anatomical measurements increase, the flat and mean keratometry of the anterior topography decrease proportionally, and the same happens with the flat and mean keratometry of the posterior surface of the cornea.

In conclusion, this study demonstrates associations between preoperative topography, including posterior topography, and the pterygium tomographic classification (nodular or flat) and its anatomical measurements assessed only by AS-OCT, which are not assessable with SLE. This information helps us to better understand the mechanism by which corneal topography is affected in patients with pterygium, with future prognostic and therapeutic implications. We propose the systematic use of AS-OCT for the initial assessment of pterygium because it would predict its astigmatic effect on the cornea and therefore its possible reduction after surgery depending on its classification as nodular or flat and its anatomical measurements. Thus, according to the conclusions of this study, the eyes that, by means of AS-OCT evaluation, present flat pterygia with higher anatomical measurements, have a greater topographic affectation and, therefore, a greater reduction in astigmatism after surgery can be assumed. This may help to guide the surgical indication of pterygium, for example, prior to cataract surgery, and to manage patient expectations regarding visual outcomes after surgery.

## Figures and Tables

**Table 1:** Total sample size (number of eyes) needed to detect a certain effect size in the degree of correlation between two continuous parameters. Calculated for 3 levels of statistical power (95% confidence level with Pearson’s linear correlation coefficient null test).

Degree of Correlation	Power Achieved		
	70%	80%	90%
r = 0.3 (weak)	67	84	112
r = 0.4 (weak-moderate)	37	46	61
r = 0.5 (moderate)	23	29	37
r = 0.7 (strong)	11	13	17

**Table 2:** Preoperative astigmatism variable comparisons by atrophic/intermediate/fleshy pattern in LH: independent samples *t*-test results.

	*p*-Value
CYL	0.310
K1	0.261
K2	0.604
AvgK	0.271
CYLp	0.509
K1p	0.443
K2p	0.575
AvgKp	0.401
CYLr	0.438
K1r	0.307
K2r	0.678
AvgKr	0.295

**Table 3:** Preoperative astigmatism type comparison by atrophic/intermediate/fleshy pattern in LH: chi-squared test results.

	*p*-Value
AIF pattern	0.815

**Table 4:** Preoperative astigmatism variable comparisons by nodular/flat pattern in AS-OCT: independent samples *t*-test results.

	*p*-Value
CYL	0.072
**K1**	0.025 *
K2	0.280
**AvgK**	0.028 *
CYLp	0.802
K1p	0.098
K2p	0.176
AvgKp	0.138
CYLr	0.060
**K1r**	0.022 *
K2r	0.307
**AvgKr**	0.024 *

* *p* < 0.05.

**Table 5:** Preoperative astigmatism axis comparison (with-the-rule, against-the-rule, or oblique) by nodular/flat pattern in OCT-SA: chi-squared test results.

	*p*-Value
NF pattern	0.855

**Table 6:** Thickness parameter comparisons by preoperative astigmatism type by axis: ANOVA (F) test results.

	*p*-Value
LimbusT	0.926
**CentreT**	0.047 *
HeadT	0.786
HorizontalCornealInv	0.789
EpitT1mm	0.361
StromT1mm	0.534
TotalT1mm	0.518
TotalT2mm	0.484
TotalT3mm	0.310

* *p* < 0.05.

**Table 7:** Multiple comparisons of the “Center Thickness” variable by preoperative astigmatism type: *t*-test results with Bonferroni correction.

	*p*-Value
With–Against	0.173
**Against–Oblique**	0.042 *
Oblique–With	0.451

* *p* < 0.05.

**Table 8:** Relationship between astigmatism parameters and preoperative pterygium thickness: Pearson correlation coefficient results.

	**CYL**	**K1**	**K2**	**AvgK**
**LimbusT T0**	0.732 (r = −0.05)	0.566 (r = −0.09)	0.660 (r = −0.06)	0.537 (r = −0.09)
**CentreT T0**	0.693 (r = 0.06)	0.910 (r = −0.02)	0.712 (r = −0.05)	0.927 (r = −0.01)
**HeadT T0**	0.314 (r = 0.15)	0.717 (r = 0.05)	0.395 (r = −0.12)	0.925 (r = −0.01)
**HorizCornealInv**	<0.001 *** (r = −0.60)	<0.001 *** (r = −0.50)	0.736 (r = 0.05)	0.017 * (r = −0.34)
**EpiT1mm T0**	0.190 (r = −0.19)	0.040 * (r = −0.29)	0.178 (r = −0.19)	0.035 * (r = −0.30)
**StromT1mm T0**	0.906 (r = −0.02)	0.216 (r = −0.18)	0.073 (r = −0.26)	0.082 (r = −0.25)
**TotalT1mm T0**	0.770 (r = −0.04)	0.149 (r = −0.21)	0.062 (r = −0.27)	0.054 (r = −0.27)
**TotalT2mm T0**	0.274 (r = −0.16)	0.056 (r = −0.27)	0.155 (r = −0.20)	0.041 * (r = −0.29)
**TotalT3mm T0**	0.122 (r = −0.22)	0.049 * (r = −0.28)	0.380 (r = −0.13)	0.071 (r = −0,26)
	**CYLp**	**K1p**	**K2p**	**AvgKp**
**LimbusT T0**	0.362 (r = 0.13)	0.905 (r = 0.02)	0.892 (r = 0.02)	0.583 (r = 0.08)
**CentreT T0**	0.914 (r = 0.02)	0.938 (r = −0.01)	0.858 (r = −0.03)	0.910 (r = 0.02)
**HeadT T0**	0.260 (r = 0.16)	0.907 (r = −0.02)	0.792 (r = 0.04)	0.652 (r = 0.07)
**HorizCornealInv**	0.597 (r = 0.80)	0.426 (r = 0.12)	0.791 (r = 0.04)	0.480 (r = 0.10)
**EpiT1mm T0**	0.497 (r = 0.10)	0.077 (r = 0.25)	0.052 (r = 0.28)	0.094 (r = 0.24)
**StromT1mm T0**	0.903 (r = −0.20)	0.044 * (r = 0.29)	0.175 (r = 0.19)	0.055 (r = 0.27)
**TotalT1mm T0**	0.987 (r = 0.00)	0.032 * (r = 0.30)	0.123 (r = 0.22)	0.042 * (r = 0.29)
**TotalT2mm T0**	0.443 (r = 0.11)	0.283 (r = 0.15)	0.194 (r = 0.19)	0.198 (r = 0.19)
**TotalT3mm T0**	0.330 (r = 0.14)	0.606 (r = 0.07)	0.521 (r = 0.09)	0.437 (r = 0.11)
	**CYLr**	**K1r**	**K2r**	**AvgKr**
**LimbusT T0**	0.579 (r = −0.08)	0.545 (r = −0.09)	0.781 (r = −0.04)	0.484 (r = −0.10)
**CentreT T0**	0,813 (r = 0.03)	0.918 (r = 0.01)	0.746 (r = −0.05)	0.920 (r = −0.01)
**HeadT T0**	0.381 (r = 0.13)	0.748 (r = 0.05)	0.355 (r = −0.13)	0.851 (r = −0.03)
**HorizCornealInv**	<0.001 *** (r = 0.60)	<0.001 *** (r = −0.51)	0.532 (r = 0.09)	0.022 * (r = −0.32)
**EpiT1mm T0**	0.164 (r = −0.20)	0.046 * (r = −0.28)	0.253 (r = −0.16)	0.033 * (r = −0.30)
**StromT1mm T0**	0.779 (r = −0.04)	0.232 (r = −0.17)	0.085 (r = −0.25)	0.081 (r = −0.25)
**TotalT1mm T0**	0.648 (r = −0.07)	0.163 (r = −0.20)	0.077 (r = −0.25)	0.053 (r = −0.27)
**TotalT2mm T0**	0.203 (r = −0.18)	0.052 (r = −0.28)	0.183 (r = −0.19)	0.032 * (r = −0.30)
**TotalT3mm T0**	0.093 (r = −0.24)	0.044 * (r = −0.29)	0.435 (r = −0.11)	0.056 (r = −0.27)

* *p* < 0.05; *** *p* < 0.001.

## Data Availability

In this study, no new data were created.
